# Effectiveness of Tele-Prescription of Therapeutic Physical Exercise in Patellofemoral Pain Syndrome during the COVID-19 Pandemic

**DOI:** 10.3390/ijerph18031048

**Published:** 2021-01-25

**Authors:** Manuel Albornoz-Cabello, Cristo J. Barrios-Quinta, Ana M. Barrios-Quinta, Isabel Escobio-Prieto, María de los Angeles Cardero-Durán, Luis Espejo-Antunez

**Affiliations:** 1 Department of Physiotherapy, Faculty of Nursing, Physiotherapy and Podiatry, University of Sevilla, 41009 Sevilla, Spain; malbornoz@us.es; 2 Physiotherapy Unit, Andalusian Health Service, 41005 Sevilla, Spain; cristo.barrios@gmail.com; 3 Nursing Unit, Andalusian Health Service, 41005 Sevilla, Spain; anam.barrios.sspa@juntadeandalucia.es; 4 Department of Medical-Surgical Therapy, Medicine Faculty, University of Extremadura, 06071 Badajoz, Spain; m.angeles.cardero@gmail.com (M.d.l.A.C.-D.); luisea@unex.es (L.E.-A.)

**Keywords:** therapeutic physical exercise, patellofemoral pain syndrome, physiotherapy

## Abstract

The health crisis caused by COVID-19 has had a huge impact on the provision of physiotherapists’ services during the pandemic. Patellofemoral pain syndrome (PFPS) is a major health problem and one of the most common causes of pain in the front of the knee in outpatients. The objective was to evaluate the effectiveness of a therapeutic physical exercise (TPE) program supervised by a physiotherapist using telematic channels in reducing pain and disability in a sample of 54 patients with PFPS in the Physiotherapy Service of the San José de la Rinconada Health Center (Seville). Subjects were evaluated pre- and post-intervention (4 weeks—12 treatment sessions). An analysis was made of perceived pain—using the visual analog scale (VAS) and the DN4 neuropathic pain questionnaire—and functional balance—through the Kujala Score test and the Lower Extremity Functional Scale. The supervised TPE program in patients with PFPS produced a reduction in pain: VAS F_1, 52_ = 8.68 (*p* = 0.005) η^2^ = 0.14 and DN4: F_1, 52_ = 69.94 (*p* = 0.000) η^2^ = 0.57; and in Lower Extremity Functional Scale (LEFS) disability: F_1, 52_ = 19.1 (*p* = 0.000) η^2^ = 0.27 and KUJALA: F_1, 52_ = 60.28 (*p* = 0.000) η^2^ = 0.54, which was statistically significant (*p* = 0.000 for *p* < 0.05). Hence, the TPE program presented was effective in reducing pain and disability in patients with PFPS.

## 1. Introduction

The period of time covering the present study is unprecedented. On 14 March 2020, the Government of Spain decreed a state of emergency, thereby beginning a period of confinement in order to stop the spread of the SARS-CoV-2 virus. This situation forced face-to-face care of patients attending the Physiotherapy Rooms in Primary Care Centers on an outpatient basis to be cancelled [1]. From that precise moment, physiotherapists in the primary care basic teams began to think of alternatives in order to maintain physical activity in their patients. Therapeutic exercise routines would make it possible to maintain the therapeutic goals of the different programs being carried out depending on their pathology.

It was a question of developing a labor of health education in the population being cared for by said physiotherapy rooms. Explaining the basic measures of prevention of the spread of the virus (hand hygiene, face masks, avoiding social contact, etc.) was necessary, as well as eliminating the techniques that would require contact, whilst at the same time providing self-management and self-care tools that matched the pathology of each patient, all of this while prioritizing telecare, recurring to telematic resources (telephone calls, emails, audio-visual resources, links to online material, etc.) [2]. Some studies have suggested a high social acceptance and confidence of patients toward telehealth in trauma care, especially for real-time diagnosis and remote treatment [3]. In addition, the specific e-Health and telemedicine programs implemented in the evaluation and treatment of musculoskeletal problems can reduce health costs. These programs may generate significant impact on patients living in rural or remote areas and increase adherence to treatment [3]. At the initial stage of the global pandemic declaration by the WHO, this was the only possibility of continuing to provide services and avoiding risks to health professionals. In this case, primary care physiotherapists, given the lack of personal protective equipment at the beginning of the pandemic, needed to suspend in-person services.

Patellofemoral pain syndrome (PFPS) is a common musculoskeletal problem, characterized by pain in the front of the knee and tending to become chronic [4]. Although it affects the whole population, its incidence is greater in adolescents and young adults [5,6].

PFPS has been associated with osteoarthritis of the knee and a high body mass index, but in a recent study, it was observed that this is not true in adolescents [7]. Patients usually describe an increase in symptoms on going up and down stairs, squatting, running, or sitting for a long time; since these activities increase compressive load forces in the patellafemoral articulation [8].

PFPS continues to be one of the most common and challenging musculoskeletal issues facing physiotherapists and sports medicine professionals [9].

Even though the pain associated with PFPS is characteristic, the cause of this pain remains unknown. Traditionally, PFPS has been related to damage of the articular cartilage; however, we know that articular cartilage is aneural [10]. There exist a variety of pathologies that can present signs and symptoms similar to PFPS, wherefore it is used to refer to all pain in the front of the knee [11].

The lack of understanding of the etiology and pathology associated with patellofemoral pain and dysfunction is reflected in the great number of therapeutic options to deal with PFPS. Conservative treatments are common, above all initially, and physiotherapy is one of the commonest interventions used [9].

Physiotherapeutic treatments often include the strengthening of the vastus medialis muscle of the quadriceps to promote active stabilization of the patella inside the trochlea of the femur, as well as manual therapy procedures, patellar realignment through taping, stretching, and therapeutic exercise [12,13,14]. Although these treatments seem to be based on theoretical reasoning, the evidence as to the effectiveness of these interventions is not well established [11].

Therapeutic physical exercise (TPE) constitutes one of the most valuable tools in our therapeutic arsenal. Adequate prescription tailored to each patient depending on their pathology, prior state of health, and prior levels of physical activity is a demand that the health system and society places on our backs. This entails the configuration of the physiotherapist as an expert in TPE. They must supervise and optimize the TPE program, adapting it to each patient, monitoring the prior and aspirational levels of physical activity in terms of both quantity and quality. In addition, the physiotherapist must identify any psychosocial barriers that might hinder the adherence of patients to the prescribed TPE programs. In short, the physiotherapist becomes the guide of the patient who must take the reins of their health and take on a proactive role in the face of their pathology [4,15].

TPE and the integration of individual and group health education, in the activity of the primary care physiotherapist, afford greater control of repetitive demands on physiotherapy in the medium-term. Hence, supervised TPE has been hailed as one of the most effective tools in resolving problems deriving from chronic conditions where the reduction of pain and disability are the main objectives to be achieved [16]. The purpose of this study is to evaluate the effectiveness of a TPE program in reducing pain and disability, to quantify the decrease in pain in the front of the knee, and to appraise the improvement in functional disability in patients with PFPS after treatment with TPE supervised by a physiotherapist via telematic channels.

## 2. Materials and Methods

### 2.1. Study Design

The present study is a longitudinal and prospective clinical trial conducted in the Physiotherapy Service of the San José de la Rinconada Health Center (Seville, Spain), dependent on the Andalusian Health Service. This study was supervised by the institutional ethics committee CEI University Hospital Virgen Macarena and Virgen del Rocio, with ethics approval number 41255cfcgab78019fga8501bb354b0ba4b15a804, registered in the Australian New Zealand Clinical Trials Registry: ACTRN 12619001457134, available at http://www.anzctr.org.au/Trial/Registration/TrialReview.aspx?id=377666&isReview=true and found to be in agreement with the Declaration of Helsinki.

### 2.2. Participants

The target population that this protocol was aimed at was made up of all patients diagnosed with unilateral or bilateral PFPS belonging to the health area of the Basic Health Area of la Rinconada (Seville), with over 36,000 potential users. Inclusion criteria were patients diagnosed with PFPS of over a 6 month evolution, aged between 18 and 70 years. The exclusion criteria were presenting cognitive alterations and having undergone conservative or surgical treatment of the affected knee less than 6 months previously.

### 2.3. Sampling and Randomization

The convenience sampling method was used based on previous studies [17], an appropriate formula for the comparison of two proportions. The total number of patients who began the study was 60 (30 + 30), and only 6 patients (3 + 3) did not finish the study (due to dropping out or not answering the appointment or supervision calls). The final estimated sample size with a 10% dropout was equal to 27 individuals in each group (α = 0.05, β = 0.2). Eligible patients were randomly divided into two groups after providing written informed consent with the epidemiological data analysis program (EPIDAT) [18] program. All of them were Caucasian subjects. The first control group (C group) received indications via telematic channels from their physiotherapist following the Spanish Society of Rehabilitation and Physical Medicine (SERMEF) indications [17], whereas the second TPE group, as well as receiving the indications via telematic channels from their physiotherapist following SERMEF indications [19], also received telephone advice at least three times a week over the four-week period that was the object of the study.

### 2.4. Dependent Variables

Measurements of the different variables of the study were made at two times, at the beginning thereof and after a four-week period, which coincided with the end of the intervention or treatment plan (12 home-based TPE sessions supervised by the physiotherapist via telematic channels).

The dependent variables considered in the heart of our research were (i) the *perception of pain* by the visual analog scale (VAS) which consists of using a validated scale, 10 cm long (0–10), where subjects place themselves from (0) lack of pain to (10) the worst pain bearable; (ii) *perception of neuropathic pain*, established through the DN4 questionnaire [20]; (iii) *functional disability*, quantified using the Kujala Score [21] and the Lower Extremity Functional Scale (LEFS) [22]; and (iv) an anthropometric study of the knee was also performed [5].

### 2.5. Protocol of Intervention

*Interventions*. Patients in the TPE group learned strengthening, endurance, flexibility, and an active range of motion exercises. A training program was created (Table 1) based on SERMEF indications [19]: above all, to activate the vastus medialis of the quadriceps muscle; to produce an authentic effective break to the lateralizing tendency of the patella during knee extension movements; as well as to work on the hamstrings, which are essential so that there is a balance of forces acting on the knee [12]; and to work on the tibia internal rotator muscles and strengthening of the triceps surae, thereby decreasing patellar pressure, owing to the posterior displacement of the lower extremity of the femur (synergy of quadriceps and triceps surae).

Then, they received a pamphlet containing descriptions and pictures detailing the above exercises (Table 1). Patients were asked to continue these exercises three times a week for four weeks (total of 12 sessions). They were told to place a cold pack (or similar) on their knees for 20 min before each session. A physiotherapist was responsible for making contact with patients via phone call and email three times a week where patients explained their exercise program and consulted their doubts about the execution. In this manner, the specialist remotely monitored the progress of the exercises and maintained principles of daily activities and symptom improvements. The physiotherapist asked patients to carry out their exercises as instructed by the pamphlet they received.

In the C group, patients performed the same exercises as the TPE group, after being instructed by the physiotherapist on the exercises to be done at home but with no phone call control.

**Figure 1:**
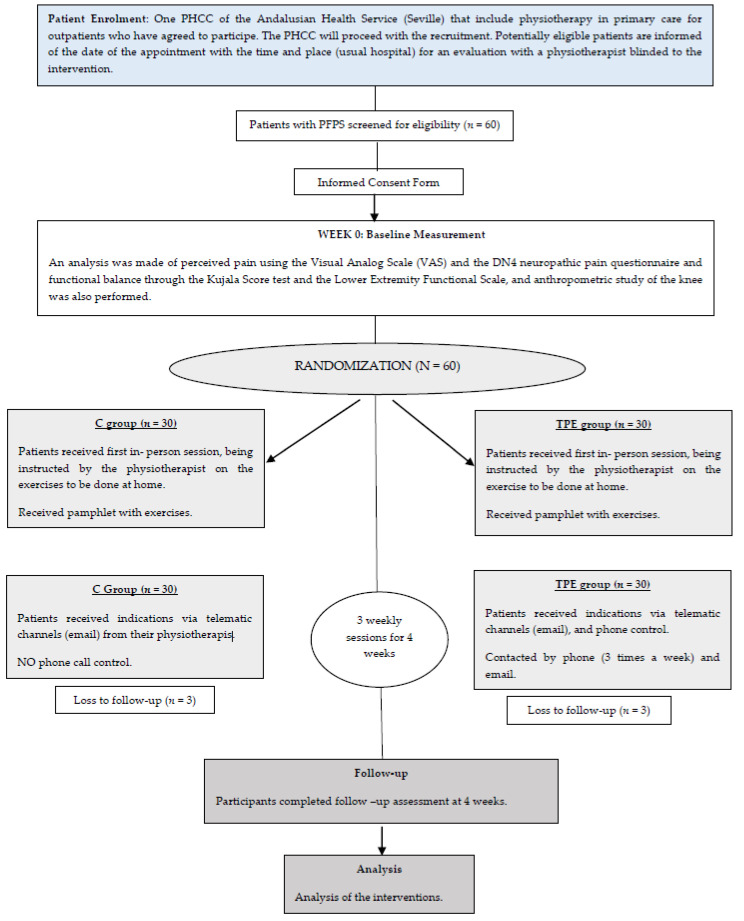
Design and flow participants through the trial. PHCC, primary healthcare centre; VAS, visual analog scale; TPE, therapeutic physical exercise group.

*Pre- and post-treatment evaluations*. In both groups, after acquiring demographic data, the intensity of knee pain was assessed with the VAS before the first session of treatments. Then, the KUJALA score, LEFS, and DN4 test were filled out by the physiotherapist to measure neuropathic knee pain, symptoms, and physical function throughout the day. Patients were also provided with X Index for evaluation of physical function. The KUJALA and DN4 scores were normalized ((acquired score/total possible score) ×100). In accordance with previous studies [17], participants were re-evaluated using the same scales at the end of four weeks to assess the effects of each treatment (Figure 1).

### 2.6. Statistical Analysis

The sample size calculation was based on the detection of (1) an improvement of 15% in self-perceived pain intensity [23] and (2) a difference of >9 points in LEFS score at inter-group comparison after the treatment [24] and >10 points in the Kujala score. Taking into account a one-tailed hypothesis, an alpha value of 0.05, a desired power of 80%, and a large effect size (f = 0.2), 26 participants were required per group of treatment (G * Power, version 3.1.9.2).

The statistical analysis of the data was carried out using PSW Advanced Statistics (SPSS Inc, Chicago, IL) version 24.0. Data were reported as mean (standard deviation) and confidence intervals (IC 95%). Firstly, the normal distribution of variables was verified by the Shapiro–Wilk test after descriptive analysis. The Levene test was used to assess the homogeneity of variances. Linearity was assessed by bivariate dispersion graphics of residual values observed from the expected values. Comparisons between groups were made for demographic and clinical data of reference using Student’s t-test for continuous variables and Pearson’s chi-square test for categorical variables. All analyses followed the intention-to-treat principle, and the groups were analyzed as randomized.

Differences in the measurements were detected by analysis of covariance (ANCOVA) to evaluate each dependent variable by age, height, weight, and body mass index, and as a variable factor the group (group TPE versus C). Eta square (η^2^) was used to calculate the effect size (small when 0.01 ≤ η^2^ ≤ 0.06; medium when 0.06 ≤ η^2^ > 0.14; large when η^2^ > 0.14). Statistical significance was determined at *p* < 0.05.

## 3. Results

### 3.1. Description of the Sample

A total of 60 subjects, aged between 25 and 67 years, were selected for the trial. After the inscription phase, the final sample included 54 individuals (*n* = 54), 26 men and 28 women; mean age (SD) was 51 years (10.1). Of the affected knees, 29 were right (54%) and 25 left (46%). Of the subjects, 24 had an educational level of primary studies (44%); 18, secondary (34%); and 12, university (22%). Of the sample subjects, 38 were employed in the service sector (82%) and 10 in construction (18%).

In relation to the patient diagnoses: 23 with patellar tendinitis (44%), 21 with gonarthrosis (40%), 7 with chondromalacia patella (12%), and 3 with bursitis (4%) took NSAIDS and analgesics, while 28 subjects (52%) took nothing.

Table 2 shows the mean values and standard deviation of the main variables, both as a whole and for each study group (experimental group and control group) and their statistical significance (*p* > 0.05), confirming that the groups had equivalent values at the beginning of the study.

Although the knee extension variable was measured by goniometric measurement, as all the knees in the study presented a value = 0°, or full extension, it is neither presented nor analyzed in the present study.

### 3.2. Analysis of Outcome Measures about Disability

Table 3 includes the initial and final scores of the variables in the study, and the differences between measurements before and after treatment.

Statistical significance was found in favor of the experimental group in the between-groups comparison and in the interaction in the covariables age, height, weight and body mass index, in both the perception of pain: VAS F_1, 53_ = 4.77 (*p* = 0.001) η^2^ = 0.33 and DN4 F_1, 53_ = 6.99 (*p* < 0.001) η^2^ = 0.42; and in the knee disability index: LEFS: F_1, 53_ = 4.38 (*p* = 0.002) η^2^ = 0.31 and KUJALA: F_1, 53_ = 10.58 (*p* < 0.001) η^2^ = 0.52; as well as, finally, in range of movement: Flexion: F_1, 53_ = 6.35 (*p* < 0.001) η^2^ = 0.50. No statistically differences were found between before- and after-treatment measurements regarding the use of basic analgesic drugs. Finally, it must be considered that no side effects were observed.

## 4. Discussion

In this study, we evaluated the effectiveness of a telematic TPE program, supervised by a physiotherapist, in reducing pain and disability in patients with PFPS. In all participants, a significant improvement was observed in pain intensity, range of movement, and disability from baseline to four weeks of treatment, according to VAS, LEFS, DN4, and KUJALA indices.

According to the most recent systematic reviews on the approach to PFPS, TPE is by far the most used therapeutic alternative [24]. Both due to its effectiveness as a treatment, and due to the ease of carrying it out in the home environment—needing few resources and given the situation of confinement that our target population was subjected to—practically no other alternative was left.

In the abovementioned reviews, the primary option is pointed out as eccentric muscle work, demonstrating a more important improvement in functional balance, as well as patient satisfaction, than other alternatives [25]. This effectiveness would be related to the increase in production of collagen at the quadriceps tendon level and correct alignment of the new fibers synthesized, thereby improving their structure. Likewise, the formation of new vessels is produced, which would lead to an attenuation of the levels of pain perceived by the nociceptive nerve fibers present in the structure. Despite the numerous studies conducted, exercise type, frequency, and load are highly variable in the different studies included in the systematic reviews [26].

This makes it absolutely essential to carry out more comprehensive studies in the future [6,27]. Meanwhile, isometric exercise has also been pointed out as one of the most interesting therapeutic alternatives to tackle PFPS. It is above all due to the analgesic effect it induces nearly immediately, which lasts for 45 min after being carried out, practically from the first few sessions. This analgesia is related to muscular inhibition at the cortical level. This makes isometric exercise a tool of great clinical usefulness. For this reason, we guide our patients to do this before and after carrying out the therapeutic exercise program, recommending that they leave the exercises that involve concentric or eccentric work or subject the knee joint to loads to the middle of the program. Thus, we make the most of this induced analgesia by using it to our advantage to boost the therapeutic adherence of our patients [4,27].

One of the limitations of our study was the relatively short-term follow-up of four weeks due to the lack of adherence to the treatment of our subjects, which in our case was limited to three subjects (who did not finish the study) per group.

Another limitation was the methods used for telecommunications, i.e., phone and email (and audiovisual resources and links to online material only if it was necessary) but not videoconference. It is obvious that by increasing the attractiveness of home-based programs, we can facilitate patient adherence to tele-rehabilitation [17].

One of the strengths of our study was the fast adaptation of the physiotherapy service to the pandemic, with low resources available, so as not to interrupt the treatment of patients. Another of the strengths is the similarity between our participants’ characteristics and those of participants in other studies. This may help other researchers to achieve a common conclusion by reviewing these study results.

## 5. Conclusions

The perceived pain in patients with PFPS after the application of a TPE protocol supervised by a physiotherapist via telematic channels was reduced on average by 1/10, measured using the VAS and DN4 scales.

Application of TPE supervised by a physiotherapist via telematic channels was effective and produced changes in the degree of disability of patients afflicted with PFPS measured through the LEFS with an increase of 15 points, rising from 45 to 60.

Measurement of this disability using the KUJALA questionnaire presented a mean reduction of 20 points, going from 49 to 69.

Finally, it was determined that range of movement was increased by an average of 9°.

It can be concluded, therefore, that therapeutic exercise supervised by a physiotherapist via telematic channels demonstrates greater effectiveness in pain reduction and disability in patients afflicted with PFPS than merely providing information sheets to the patient.

## Figures and Tables

**Table 1:** Training program. Patellofemoral pain syndrome basic plus stretches.

Exercise	Starting Position (sp)	Execution	Explanation
**straight-leg lift**	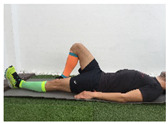	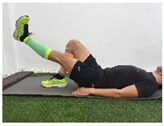	Raise straight leg with knee in extension about 30 cm from the ground. Hold for 5” and slowly return to SP. Sets (S): 1/Repetitions (R): 5
**supine knee extension (last 30°)**	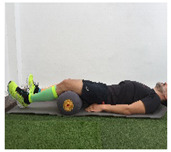	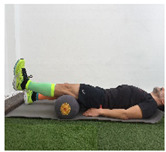	Lie on your back with a ball under your knee for support. Straighten your knee, keeping contact with the ball. Ankle must be at 90°. Hold for 5” and return to SP. S:1/R:10
**supine knee extension against ball**	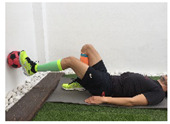	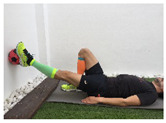	Lie on your back, keeping your knee slightly flexed while you hold a ball against the wall. Press the ball. Hold for 5” and then relax. S:1/R:10
**wall squat**	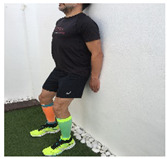	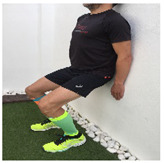	Lean against the wall with your back. Bend your knees and slide down the wall until your knees are 30–45° flexed (according to pain tolerance). Hold this position for 2–3” and quickly return to the SP. S:3/R: 10
**lateral step**	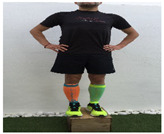	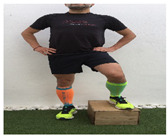	Lower your non affected leg, sideways, down to the ground, slowly flexing the affected leg (no more than 45°). Back to SP doing full leg extension. S:1/R:10
**standing knee extension with resistance band**	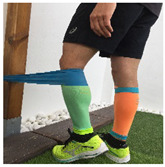	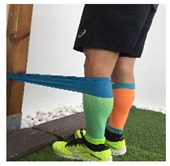	Stand in front of a table, with a resistance band tied around your knee and the table leg. Straighten your knee until your heel reaches the floor. Hold for 5” and return to SP. S: 1/R:10
**standing quadriceps stretch**	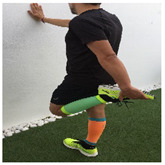	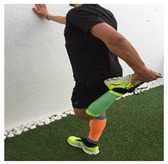	Stand on one leg leaning on a wall. Bend your knee and hold your foot with your hand. Passively bring the heel toward your buttock until a stretch is achieved. Hold for 10–30”and release. S: 1/R: 5
**standing hamstring stretch**	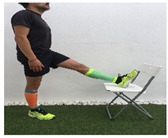	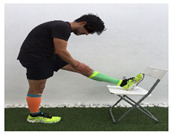	Tilt the body forward preventing the knee from bending and keeping the lumbar spine straight. Hold it for 10–30”. S:1/R: 5
**standing triceps surae stretch**	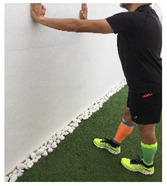	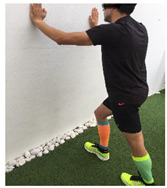	Stand leaning into the wall, with the affected foot behind the non-affected one. Bend your elbows and the non-affected knee, keeping your affected knee straight and heel on the floor, until a stretch is achieved. Hold for 10–30”and the release. S: 1/R: 5
**sitting iliotibial band stretch**	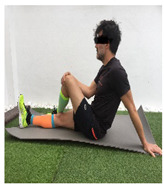	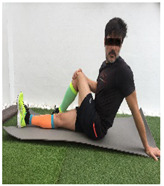	Sit on the floor with the affected leg crossed over the other one. Turn your torso to the affected side while you pull your affected knee up towards the opposite shoulder, until a stretch is achieved. Hold for 10–30” and release. S: 1/R: 5

**Table 2:** Baseline characteristics of study groups.

	Total Sample (*n* = 54)	TPE Group (*n* = 27)	C Group (*n* = 27)	*p*-Value *
Mean age (years)	51 (10.1)	51 (11.0)	51 (9.37)	0.979
Height (cm)	167 (11.0)	169 (11.7)	165 (10.0)	0.176
Weight (kg)	81 (16.8)	82.8 (17.8)	79.1 (15.8)	0.411
Body Mass Index	28.7 (4.8)	28.6 (4.3)	28.9 (5.4)	0.827
LEFS (%)	46 (12.4)	45 (15.2)	47 (8.9)	0.530
KUJALA (%)	49 (14.2)	49 (14.9)	50 (13.7)	0.865
DN4 (0–10)	3.8 (1.61)	3.7 (1.2)	3.8 (1.9)	0.933
VAS (mm)	61 (15.2)	58 (12.1)	63 (17.6)	0.169
FLEXION (°)	118 (12.3)	117 (11.0)	119 (13.5)	0.548

Data are reported as mean (SD); * between-groups statistical significance (one-factor ANOVA).

**Table 3:** Baseline, post-intervention, and mean score changes of knee pain and lower extremity function.

	Baseline	Post-Intervention	Within-Group Mean Changes	Between-Groups Mean Changes
LEFS				
Control TPE	47 (8.9) 45 (15.2)	50 (10.3) 60 (9.0)	3 [1/5] 15 [9/19] **	10 [3/14] ^†^
KUJALA				
Control TPE	50 (13.7) 49 (14.9)	53 (12.5) 69 (13.1)	3 [1/5] * 20 [16/23] **	16 [8/22] ^††^
DN4				
Control TPE	3.8 (1.9) 3.8 (1.3)	4 (1.9) 1.9 (1.1)	0.2 [0.1/0.4] 1.9 [1.3/2.3] **	2.1 [1.1/2.9] ^††^
VAS				
Control TPE	63 (17.6) 58 (12.1)	63 (18.8) 48 (13.1)	0 [−2/3] 10 [3.7/15] *	15 [5/23] ^†^
FLEXION				
Control TPE	110 (16.2) 117 (11.0)	111 (16.5) 126 (12.3)	1 [−3/2] 9 [5/13] **	15 [4/24] ^†^

LEFS: Low extremity functionality scale; DN4: Neuropathic pain in four questions; VAS: visual analogue scale. Data are reported as mean (SD) or (95% confidence level); * indicates statistically significant within-group differences (*p* < 0.05); ** indicates statistically significant within-group differences (*p* < 0.001); ^†^ indicates statistically significant within-group differences (*p* < 0.05); ^††^ indicates statistically significant between-groups differences (*p* < 0.001).
